# Physiological Regulation of Innate Lymphoid Cells

**DOI:** 10.3389/fimmu.2019.00405

**Published:** 2019-03-12

**Authors:** Nicolas Jacquelot, Kylie Luong, Cyril Seillet

**Affiliations:** ^1^Molecular Immunology Division, Walter and Eliza Hall Institute of Medical Research, Melbourne, VIC, Australia; ^2^Department of Medical Biology, University of Melbourne, Melbourne, VIC, Australia

**Keywords:** innate lymphoid cells, immunity, homeostasis, neuroendocrine regulation, metabolites, hormones, neuropeptides

## Abstract

Discovery of innate lymphoid cells (ILCs) have provoked a paradigm shift in our understanding of the immune protection. Their constitutive presence and activity at the body's barrier surfaces ensure the maintenance of the tissue homeostasis and immune protection. This complex family has distinct and non-redundant functions that can have both beneficial and detrimental effects on disease outcome. The capacity of ILCs to perform their function effectively relies on their ability to sense and integrate intrinsic and extrinsic signals. Recent studies have shown that ILCs are not only sensitive to pathogen-derived stimuli but are also very well equipped to sense host-derived signals such as neuropeptides, hormones, and metabolites. The integration of these signals represents a complex and constant cross-talk between the immune system and the physiological systems of the body, including the nervous, endocrine, digestive, and reproductive systems. The physiological regulation of ILCs constitutes an important step in our understanding of the events leading to the protective and pathological properties of these cells. This review summarizes the recent advances in the understanding of the regulation of ILCs by physiological signals and their consequences on the maintenance of tissue homeostasis.

## Introduction

With the discovery of an innate counterpart of the T lymphocytes mirroring key aspect of their phenotype and function, the innate lymphoid cells (ILCs) have forced immunologists to rethink the immunological architecture that confers immune protection. Despite recent evidence that ILCs can be mobilized from blood (1, 2), ILCs are considered to mainly reside within tissues (3). Their activity is not modulated by antigen-specific receptors but rather through a complex integration of cytokines, alarmins, and physiological signals derived from their micro-environment.

**Figure 1:**
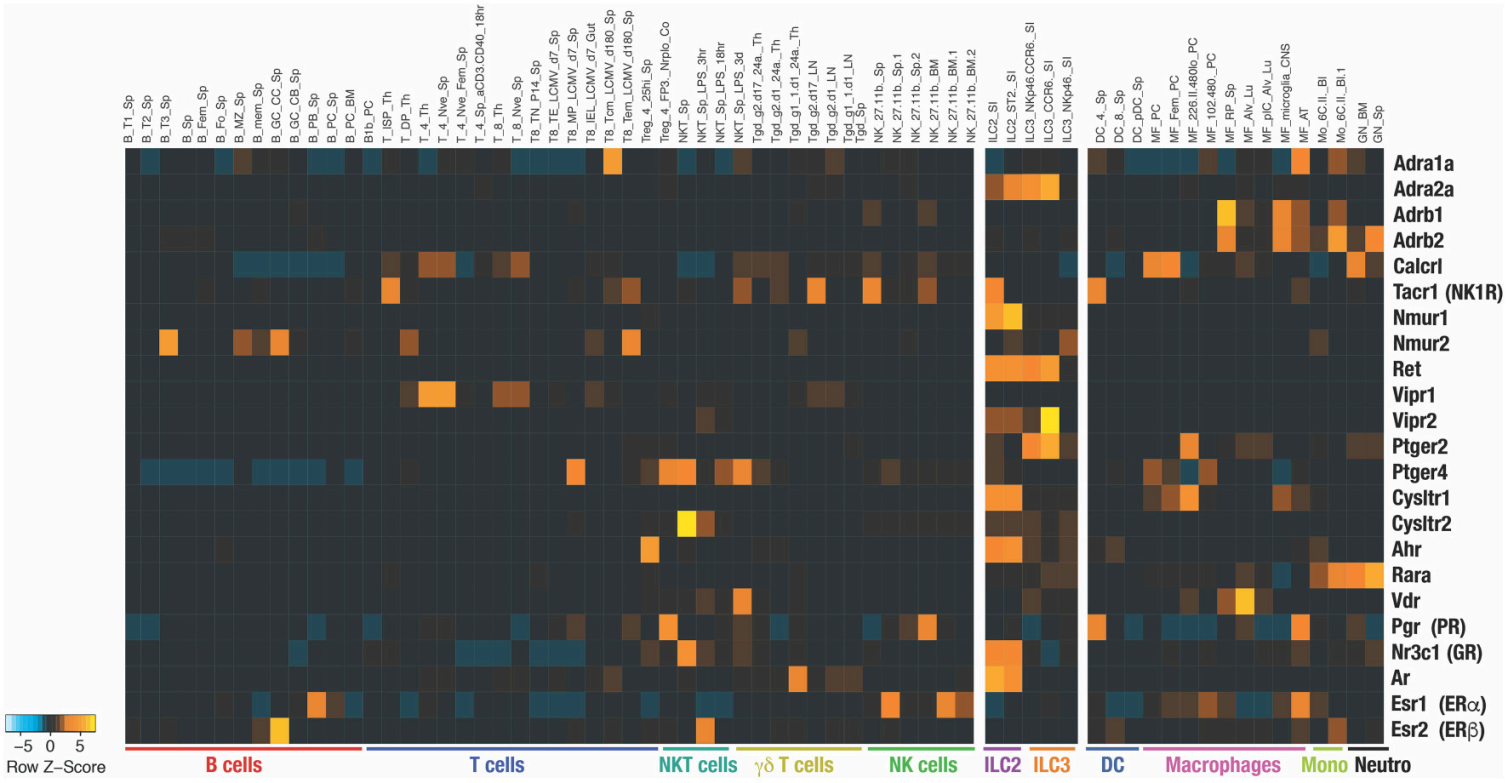
Heatmap shows the RNA expression of the indicated receptors among different immune populations. The data have been extracted from Immunological Genome Project (https://www.immgen.org) (4). Name of each population extracted from ImmGen database are indicated on top of the heatmap.

Divided in 3 main groups, group 1 ILCs (ILC1s), group 2 ILCs (ILC2s), and group 3 ILCs (ILC3s) are associated with T helper (Th) 1, Th2, and Th17 functions, respectively, while the natural killer (NK) cells are analogous to the CD8^+^ cytotoxic T cells. ILCs express particular sets of receptors encoded by specific transcriptomic signatures that are imprinted in a tissue-specific manner and therefore ILCs are well equipped to sense host-derived signals (Figure 1) (5). Constitutive sensing and integration of these endogenous signals are essential to ILC activity and maintenance of tissue homeostasis. Dysregulation of ILC responses lead to the development of inflammation. ILC1s are mainly involved in the early protection against virus (6) and bacteria (7, 8) through the secretion of interferon-gamma (IFN-γ) and granulocyte-macrophage colony-stimulating factor (GM-CSF), however their dysregulation in adipose tissues leads to the development of metabolic disorders and obesity (9). ILC2s are an early source of interleukin (IL)-5 and IL-13 (10–12). ILC2 activity allows the emergence to type 2 immune responses characterized by goblet cell differentiation, recruitment of eosinophils, basophils, and mast cells which is critical for protection against infection with helminths and viruses but, when uncontrolled, also drive allergic responses and metabolic disorder (13–15). ILC3s produce IL-22 in the gut to protect against intestinal inflammation (16–18). In this review we propose that physiological signals are integrated by ILCs and modulate their constitutive activity in a tissue- and time-specific manner.

## Neuro-Endocrine Regulation of ILC Responses

ILCs predominately reside at mucosal barrier surfaces and have been shown to be in close anatomical proximity to neurons and glial cells of the autonomic nervous system (ANS). The ANS is divided into (i) the sympathetic nervous system (SNS), which predominates during “fight-or-flight” situations and prepares the body for physical activity and (ii) the parasympathetic nervous system (PNS), which regulates basic body functions during resting conditions, such as digestion, energy conservation, and storage. The expression of specific neuroregulators by the ANS allows tissue-specific regulation of innate cells.

### Neuromedin U (NMU)

**Figure 2:**
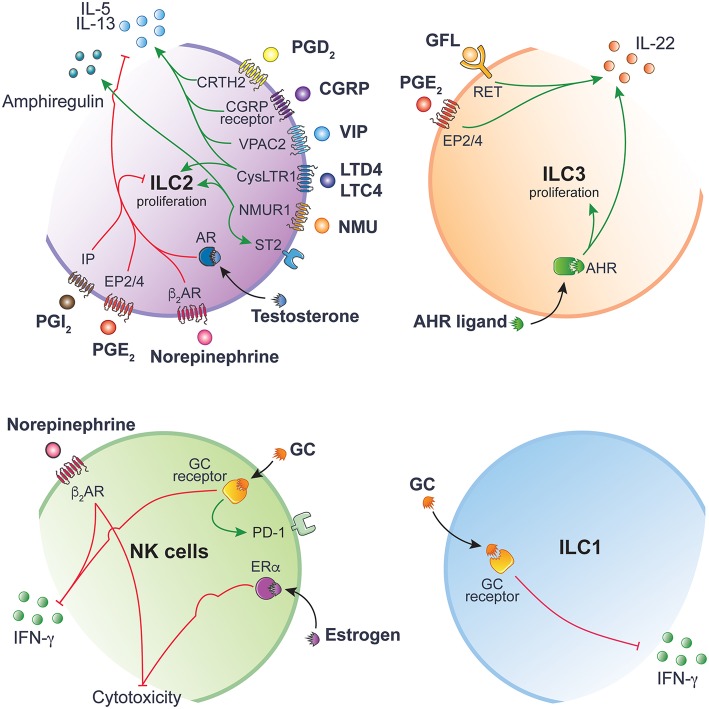
ILC function is tightly regulated by ligand-receptor interactions. Inhibitory and activating intracellular pathways are colored in red and green, respectively. This figure has been drawn using Servier Medical Art (https://smart.servier.com) and modified by the authors under the following terms: Creative Commons Attribution 3.0 Unported License.

Neuromedin U (NMU) is a neuropeptide found throughout the body and is highly expressed in the gastrointestinal tract by lamina propria enteric cholinergic neurons (19). NMU plays a plethora of physiological roles, including regulating food intake (20). NMU signals through two receptors, (i) *Nmur1*, mainly expressed on peripheral tissues, and (ii) *Nmur2*, mainly expressed within the central nervous system (CNS). Amongst immune cells, *Nmur1* is specifically expressed on ILC2 (19, 21, 22). Infection with parasitic helminth *N. brasiliensis* is sensed by mucosal neurons, inducing the secretion of NMU in response, which in turn promotes the secretion of IL-5, IL-13, and amphiregulin by ILC2s (19, 21). Deficiency in NMUR signaling leads to impaired type 2 responses and poor control of worm infection. Parasite expulsion critically relies on ILC2 activity through the specific recruitment of eosinophils, basophils, and mast cells and the induction of goblet cell hyperplasia (10) (Figure 2).

In the lung, synergistic effects of IL-25 and NMU promote ILC2 proliferation and the secretion of IL-5 and IL-13, resulting in exacerbated allergic inflammation (19, 22). Mice lacking *Nmur1* show reduced ILC2 numbers after house dust mite (HDM) challenge and decreased type 2 allergic airway inflammation (19, 22). Altogether, these studies show how neuronal cues can shape ILC responses to generate a rapid and optimal immune response.

### Norepinephrine and β2-Adrenergic Receptor Signaling

Norepinephrine is released by the SNS and signals through β_1_- and β_2_-adrenergic receptors (β_2_AR). NK cell cytotoxicity and expression of IFNγ, granzyme B and perforin are reduced when β_2_AR-signaling is engaged (23, 24).

Norepinephrine signaling exerts its inhibitory effect on ILC2s proliferation through binding to β_2_AR (25) (Figure 2). Administration of β_2_AR agonist Clenbuterol during *N. brasiliensis* infection inhibits ILC2 effector functions, leading to reduced eosinophil recruitment, goblet cell hyperplasia and consequently increased worm burden. Conversely, mice lacking the β_2_AR show increased ILC2 infiltration and exaggerated type 2 response to *N. brasiliensis* infection. Interestingly, β_2_AR-signaling specifically inhibits lung and enteric ILC2s, but not Th2 cells, by regulating cell intrinsic proliferation during type 2 inflammatory response (25).

### Vasoactive Intestinal Peptide (VIP)

VIP is a neuropeptide expressed throughout the nervous system and has been found in neurons that innervate the lung and gut mucosa (26). VIP is involved in number of physiological processes, including coordinating gastrointestinal motility, mucus, and enzymatic secretions in response to feeding, synchronizing the central circadian rhythm (27) and also skews the differentiation of T cells toward Th2 and T regulatory cells (28, 29). Enteric and lung ILC2s stimulated with VIP through VIP receptor type 2 (VPAC2) promotes a type 2 response. The circadian release of VIP in response to feeding induces a rhythmic expression of IL-5 by ILC2s (Figure 2), resulting in increased systemic eosinophil numbers in a circadian manner (30).

IL-5 stimulates the production of VIP by acting directly on nociceptors, creating an inflammatory signal loop that promotes allergic inflammation (31). Noxious environmental respiratory stimuli, such as capsaicin or OVA peptide challenge, induces bronchial hyperresponsiveness and airway inflammation through the activation of lung NaV1.8^+^ nociceptor. Ablation of NaV1.8^+^ nociceptor reduces the activation of lung resident ILC2 and Th2 cells, thus reducing bronchial hyperresponsiveness. Administering VPAC2 antagonist leads to decreased ILC2 activation, decreased expression of inflammatory marker ST2 and decreased production of IL-5 and IL-13 (31) (Figure 2). This positive feedback loop between sensory nociceptors and ILC2s may be a mechanism to prime and enhance the sensitivity of sensory nociceptors to environmental stimuli.

### Calcitonin Gene-Related Protein (CGRP) and Neurotransmitter Gamma-Aminobutyric Acid (GABA)

CGRP is a neuropeptide involved in nociception but also a potent vasodilator found throughout the body in perivascular innervation (32). During an immune response, CGRP is secreted by specialized epithelial cells called pulmonary neuroendocrine cells (PNECs). PNECs are closely associated with lung ILC2s and amplify ILC2-mediated type 2 airway inflammation in response to environmental allergens (33). In CGRP receptor-deficient mice (CalcrlKO), immune cell infiltration is reduced after HDM allergen challenge. Similarly, mice lacking PNECs (AsclCKO) show blunted type 2 immune response to ovalbumin (OVA) peptide allergen. However, when compared to control lungs, AsclCKO lungs show pronounced reduction in CGRP and inhibitory neurotransmitter gamma-aminobutyric acid (GABA). Whilst disrupting neurotransmitter GABA synthesis in PNECs does not directly affect immune response, blocking GABA signaling prevents overproduction of mucus and IL-13 and reduce goblet cell hyperplasia during allergic airway inflammation (34). As asthmatic patients have a higher number of PNECs, disrupting the communication between PNECs and ILC2s may be a potential therapeutic avenue to alleviate allergic asthma symptoms (Figure 2).

### Neurotrophic Factors and RET Receptor

Enteric ILC3s respond to neurotrophic factors released by closely associated mucosal glial cells (35). Glial cells are support cells to neurons and enteric glial cells which sense the commensal products and alarmins in a Myd88-dependent manner (35). Glial-derived neurotrophic factor family of ligands (GFL) act though ILC3 tyrosine kinase receptor RET to maintain gut defense. RET signaling in ILC3s directly controls innate IL-22 expression (Figure 2). When neurotrophic receptor RET is deleted in enteric ILC3s, ILC3-dervied IL-22 is reduced at steady state and during *C. rodentium* infection. Mice without RET showed greater propensity toward gut inflammation, leading altered microbial communities (35).

### Glucocorticoids (GC)

The hypothalamic-pituitary-adrenal (HPA) axis is a crucial neuroendocrine regulatory axis for coordinating and responding to environmental cues. GC have potent immunosuppressive and anti-inflammatory effects. Endogenous GC production after stimulation of the HPA axis inhibits ILC1 function during both bacterial (36) and viral infections (37), thus, preventing immunopathology without impairing immune responses against infections. Whilst NK cells and ILC1 are sensitive to GC during the priming phase, only NK cells from the spleen and liver show higher levels of IFN-γ production in the absence of GC receptor (GR) signaling (36). The disparity in GC influence on ILC1 suggests that the mechanisms controlling IFN-γ production in ILC1 differ from NK cells during endotoxin tolerance. When GR is deleted in ILC1 and NK cells, mice show exacerbated endotoxin LPS-induced septic shock. Similarly, GC produced following MCMV infection is shown to induce *de novo* expression of checkpoint inhibitor programmed death-1 (PD-1/CD279) specifically on splenic NK cells but not in the liver NK cells or ILC1 (37). Expression of PD-1 limits IFN-γ production and is required for the protection of the host against necrotic and granulomatous inflammation. The tissue-specificity of GC-signaling on ILC1 and NK cells results from the integration of different cytokine signals within the tissue microenvironment, allowing the fine tuning and tailoring of the immune response during infection (Figure 2).

### Estrogens

Estrogens are synthesized from cholesterol mainly in the ovaries and can diffuse through the membrane of cells and bind to their receptors in the cytosol. They can bind to the estrogen receptor (ER) α and β or act through “non-genomic” action, via modulation of intracellular signaling pathways (38). Some discrepancies exist regarding the described impact of estrogens on NK cells. While estrogen is shown to enhance the cytotoxicity of human NK-like cell line *in vitro* (39), others have reported that estrogen decreased the NK cell proliferation, cytotoxicity, and IFNγ production (40, 41). These conflicting effects of estrogens need to be carefully interpreted as the concentration used in these studies are greater than the physiological concentrations and therefore, may not reflect the actual effects of estrogens *in vivo*. As estrogens have multiple targets *in vivo*, it will be important to develop new models in which ER will be specifically deleted in NK cells to investigate the role of estrogens on this particular cell type.

Uterine ILC2 uniquely express ERα while ILC2 in other tissues do not (42). This specific pattern may explain why only uterine ILC2 have been shown to be sensitive to estrogen regulation (43). Deficiency in estrogen signaling using either ERα^−/−^ and β^−/−^ mice, or in ovariectomized mice, revealed a decreased ILC2 numbers in the uterus. The role of uterine ILC2s in regulation and maintenance of the uterus homeostasis is not known and the consequences of the regulation of these cells by estrogens will require more investigation.

### Androgens

The androgens such as testosterone and dihydrotestosterone mediate their effect via the androgen receptor (AR) and act as ER through genomic and non-genomic pathways. Regulation of ILCs by androgen signaling has been highlighted in ILC2. In mice, proportions and absolute numbers of ILC2 are greater in females compared to males (43). A similar trend is observed in humans, where female asthmatic patients have higher frequencies of ILC2s in the blood than asthmatic men (44). ILC2 progenitors express specifically the AR but not ER. Mechanistically, AR signaling directly inhibits the differentiation and proliferation of mature ILC2s in a cell intrinsic manner (43). IL-5 and IL-13 production is reduced in males compared to females and in testosterone stimulated ILC2s. This regulatory effect of testosterone on ILC2s protects males against the effects of allergen-induced lung inflammation when mice are challenged with house dust mite (HDM), IL-33 or *Alternaria alternata* (43, 44) (Figure 2).

## Metabolites Derived Regulation

There is constant communication at the mucosal barriers between the environment and the host and considerable efforts are currently made to better understand the interplay between the physiological systems and how key molecules, such as aryl hydrocarbon receptor (AHR) ligands and lipids influence local immunity and gut homeostasis.

### Aryl Hydrocarbon Receptor Ligands

AHR acts as a toxin sensor and binds to diverse endogenous and exogenous chemicals. The food and gut ecosystem are natural sources of AHR inducers. These include indoles directly found in cruciferous vegetables or obtained following tryptophan degradation by the gut microbiota (45, 46). Gut resident ILCs highly express AHR (Figure 1). The lack of AHR is associated with reduced ILC3 numbers and decreased ILC3-derived IL-22 (47, 48) (Figure 2). AHR directly binds to the *Il22* promoter and act in concert with RORγt to induce *Il22* expression in ILC3 (49).

The regulation of AHR signaling is tightly controlled as prolonged activation can have detrimental effects (50). The metabolic clearance of AHR ligands is mediated by the cytochrome P4501 (CYP1) family enzymes. Interestingly, constitutive expression of CYP1 enzymes drastically reduces the availability of AHR ligands and lead to the loss of gut ILC3 and Th17 cells (50). Consequently, constitutive *Cyp1a* expression or complete loss of *Ahr* in mice increases their susceptibility to *C. rodentium* associated with impeded IL-22 production (48–50). These studies highlight how homeostatic regulation of the availability of AHR ligands by intestinal epithelial cells provide critical feedback to immune cells, thus shaping mucosal protection.

### Leukotrienes

Leukotrienes (LT) are derived from the catabolism of the arachidonic acid. The cysteinyl leukotriene receptor 1 (CysLTR1), one of the LT receptors, is highly expressed on lung ILC2s (51, 52). LTD_4_ induces ILC2 secretion of IL-4, IL-5, and IL-13 through CysLT1R engagement (Figure 2). Administration of LTC_4_ and LTD_4_ *in vivo* synergizes with IL-33 to activate lung ILC2 and promote lung ILC2 proliferation, IL-5 expression and lung eosinophilia (52). Deficiency in LT receptors does not affect the maintenance of ILC2 but impairs the functional response of ILC2 during *N. Brasiliensis* or *Alternaria* species infection (51, 52). Human ILC2 function is also enhanced upon LT stimulation resulting in higher IL-13 production and increased expression of IL-33/IL-25 receptors, thus promoting their responsiveness to these cytokines (53). Collectively, these results demonstrate the role of LTs in promoting lung inflammation and type 2 responses through the direct activation of lung ILC2. Given the role of ILC2s in allergy and asthma, targeting this LT-CysLTR pathway is of great interest and may provide an effective therapeutic strategy to constrain ILC2-mediated inflammation (52, 54, 55).

### Prostaglandins

Prostaglandins (PG) are also derived from the arachidonic acid and exhibit different roles on ILCs. Whilst PGE2 and PGI2 signaling inhibit ILC functions (56, 57), PGD_2_ promotes the activation, migration, and accumulation of ILC2 in inflamed lung (58–60). PGE_2_ and PGI_2_ signal through the PGE_2_ receptors and the prostacyclin receptor (IP), respectively. IP is almost exclusively expressed by ILC2, PGE_2_ receptor 2 (EP2) is mainly expressed by ILC3 and PGE_2_ receptor 4 (EP4) is found on ILC1, NK cells, and ILC2 (Figure 1).

The PGD_2_ receptor, CRTH2, was first identified on Th2 cells (61) and is now widely used to distinguish human ILC2 from other ILC subsets (62). PGD_2_ in lungs amplifies type 2 immunity synergistically with IL-33/IL-25 stimulation, subsequently enhancing ILC2-derived IL-13 and chemotaxis (59, 63). This effect can be prevented by lipotoxin A_4_ in human ILC2 (63) highlighting the complex interactions between these molecules. In pathological conditions, CRTH2-deficient ILC2s do not accumulate in inflamed lungs and IL-4 and IL-13 production is impaired (58) highlighting the importance of the PGD_2_-CRTH2 axis in ILC2 regulation and control of lung inflammation and allergic disease exacerbations (Figure 2).

IP mainly act as a negative regulator of ILC function. Zhou et al. have demonstrated the negative impact of PGI_2_ on ILC2 functions (57). ILC2 stimulated with IL-33 and a PGI_2_ analog, cicaprost, show reduced cell proliferation and IL-5 and IL-13 productions (57). When IP-deficient mice are challenged intranasally with *Alternaria alternata* extracts, ILC2s accumulate in the lung and cells show enhanced IL-5 and IL-13 expression, resulting in increased eosinophils infiltration and lung inflammation (Figure 2). Similarly, PGE_2_ abrogates IL-33-induced ILC2 proliferation and cytokine production in mice (56) and humans (64). Deletion of EP4 exacerbates lung inflammation associated with ILC2-mediated eosinophil recruitments and increased ILC2-derived IL-5 and IL-13 in response to *Alternaria alternata*. Taken together, these studies reveal an evolutionarily conserved role of PGE_2_-EP2/4 pathway in negatively controlling ILC2 activity (Figure 2).

Recently, a physiological role of PGE_2_-EP4 signaling in activating the ILC3/IL-22 axis has been described. Suppression of PGE_2_ synthesis with indomethacin leads to the development of LPS-induced systemic inflammation and septic shock, resulting in elevated serum TNFα and IL-6 levels, increased spleen weight, translocation of gut bacteria and accumulation of neutrophils in the peritoneal cavity (65). LPS-induced systemic inflammation is prevented by using EP4 agonists. PGE_2_ contributes to systemic inflammation through acting on the homeostatic production of IL-22 by ILC3 (Figure 2). Suppressing PGE_2_ by indomethacin administration inhibits IL-22–IL-22R signaling pathway in intestinal epithelial cells, which lead to the downregulation of critical proteins involved in mucosal integrity such as RegIIIβ, RegIIIγ, Fut2, mucins, and molecules forming tight junctions (65). This study highlights the critical role of physiological mediators that contributes to the crosstalk between the innate immune system, gut epithelium and microflora, which confers optimal protection against systemic inflammation.

## Conclusion

The studies summarized in this review reveal a direct influence of the neuroendocrine system and metabolites on the different ILC subsets at barrier surfaces to maintain tissue homeostasis and appropriate responses during infection. They collectively demonstrate the complex interactions between neuropeptides, hormones and metabolites with ILCs and offer new opportunities to manipulate ILC responses in disease and allergy. Modulating these physiological pathways may present less side effects than synthetic drug-based strategies. Because of the synergistic or antagonistic effects between these mediators, it will be important to explore these regulatory pathways in models where the mediator or its receptor(s) can be deleted in a spatio-temporal manner. The recent ILC transcriptional analyses and database made available by Immgen Consortium have highlighted the capacity of these cells to sense a myriad of physiological signals and it will be now crucial to discover how ILCs integrate these signals to fine tune the immune response to prevent immunopathology without impairing infection control.

## Author Contributions

All authors contributed to the manuscript and read, edited, and approved the final manuscript.

### Conflict of Interest Statement

The authors declare that the research was conducted in the absence of any commercial or financial relationships that could be construed as a potential conflict of interest.
